# Walter Hubert Lecture, Towards a strategy for the detection of industrial carcinogens.

**DOI:** 10.1038/bjc.1981.188

**Published:** 1981-09

**Authors:** E. D. Acheson


Br. J. Cancer (1981) 44, 321

Walter Hubert Lecture, delivered 13 April to the 22nd AGM of the British Association for Cancer Research

### TOWARDS A STRATEGY FOR THE DETECTION OF INDUSTRIAL CARCINOGENS

E. D. ACHESON

*From the MRC Environmental Epidemiology Unit, Southampton General Hospital, Southampton SO9 4XY*

ALMOST EXACTLY six years ago, Sir Richard Doll celebrated the bicentenary of Percival Pott's publication of his account of the chimney sweep's cancer, in the seventh Walter Hubert Lecture entitled "Pott and the prospects for prevention" (Doll, 1975). Its essence was to emphasize the role which has been played by observant clinicians and pathologists in the discovery of carcinogenic agents active in man and to suggest ways in which a more systematic search might be made in future for undisclosed occupational carcinogens. In this lecture I take up a similar theme and try to deal with 4 questions:

(1) Are there likely to be as yet undisclosed occupational carcinogens?

(2) Are they worth looking for?

(3) How should we look for them? And in hard times

(4) How may we make old tools sharper and more serviceable?

### *Undisclosed occupational carcinogens*

No one who has studied the history of occupational carcinogenesis can escape the conclusion that, even allowing due credit for the brilliance of individual investigators, special situations, serendipity and luck have played a prominent role in the discovery of carcinogens to which men and women have been exposed in their places of work. For example, nasal cancer in furniture workers would probably not have been noticed had not the presence of the Chiltern beech woods with their ready supply of wood for the manufacture of chairs convenient for the London market led to the development of a large concentration of the furniture industry employing several thousand men in and around one small town in Buckinghamshire, namely High Wycombe. The fact that this town was later chosen as the site for a new district general hospital meant that a steady stream of cases of nasal adenocarcinoma was referred to a single clinic for diseases of the ear, nose and throat and seen by two surgeons, Miss Esme Hadfield and Mr Ronald Macbeth (Macbeth, 1965). If the furniture industry had been dispersed in London and other large urban centres the relationship of nasal cancer to the inhalation of hardwood dust would probably have remained undetected.

The discovery of the carcinogenic action of a factor in leather dust also leading to nasal cancer was due to two lucky chances. The first was that Northamptonshire, which contains a major part of the British footwear manufacturing industry, happens to be within the geographical area covered by the same cancer register as High Wycombe. Thus, when the regional survey of nasal cancer was carried out it disclosed not only a concentration of cases in Buckinghamshire associated with the furniture industry but a cluster in Northamptonshire where no furniture is made. Subsequently, it was shown that the Northamptonshire cluster was due to exposure (usually in men) to leather dust from soles and heels in the preparation and finishing departments of footwear factories (Acheson *et al.*, 1970; Acheson, 1976).

If the organization of the National Health Service had placed Northamptonshire in the Trent region instead of the Oxford region there is no reason to suppose that nasal cancer in the footwear industry would ever have been looked for—or found. Much later we realised that luck had helped in another way. The manufacture of men's shoes by the welting process is a special tradition of the Northamptonshire boot and shoe industry. The operations in shoemaking which create dust mostly occur during manufacture by the welting process. The risk would have been much more difficult to detect in areas of the country where a population of similar size makes other types of footwear.

Another aspect of the effects of known occupational carcinogens is that many of them cause tumours which are bizarre—or, in other words, their incidence in the unexposed populations is so low that the occurrence of 2 or 3 cases in one factory or locality is likely to be noticed. In addition to the examples I have already quoted, mesothelioma of the pleura and peritoneum, angiosarcoma of the liver, and many of the peculiarly located or multiple cancers of the skin associated with occupation (including cancer of the scrotum) come under this heading.

The action of most of the carcinogenic agents known to operate in man has been discovered in the workplace. It is difficult to imagine that there should be a special providence guiding the discovery of occupational but not other causes of cancer. It also seems unlikely that carcinogens in the workplace should usually involve large relative risks, and seldom smaller ones. On the basis of both arguments it seems likely that there may be as yet undisclosed carcinogens to which men and women have been exposed in their places of work, and that the bizarre are prominent among the occupationally related tumours detected so far because the means of detecting the more commonplace against the background are currently so poor. In other words there may be undisclosed carcinogens in the workplace which have increased the incidence of the commoner tumours and which might be revealed by systematic search.

*Are they worth looking for?*

Although evidence on which it is possible to make a precise estimate is not available, it is probable that cancer associated with exposure to carcinogens in the workplace has so far represented a small proportion of the total burden of sickness and mortality due to malignant disease. Such factors as smoking and, taken together, the consumption of alcoholic beverages, sexual habits and exposure to ultraviolet light have, up to the present, been more important. Epidemiological evidence, including for example comparisons of the incidence of cancer in different countries and in migrants between them, and studies of the incidence of cancer in persons who restrict their diet for religious and other reasons, suggest that habits of eating also exert a profound influence on the incidence of cancer (Doll, 1979).

Even if some recent estimates are accurate (Higginson & Muir, 1979) and no more than 5% of cancer in men, and a smaller proportion in women, have up to the present been occupational in origin, there are several reasons why a systematic search for further carcinogens in the workplace is important. The first reason is that the means are often more readily to hand and the opportunity for further epidemiological studies more favourable in relation to occupational factors than for studies which attempt to unravel the effects of human diet. In other words, it may often be simpler to determine "who worked where" than "who ate what" 10, 20 or even 30 years ago.

The second reason is that if a carcinogen is detected in the workplace, the measures required to prevent future cases may be relatively easy to put into effect. Thus, in the United Kingdom at any rate, it has been possible substantially to reduce the incidence of bladder cancer associated with exposure to $\beta$-naphthylamine and benzidine in the dyestuffs industry, and to control crocidolite asbestos to an extent which will reduce the incidence of mesothelioma.

A third reason is that every carcinogen that can be added to the short list of factors known for certain to exert a carcinogenic action in man is likely to help in the efforts to find valid *in vitro* and *in vivo* methods of screening new chemicals. Quite apart from any practical consideration, it would be of considerable theoretical interest to know, for example, whether such substances as ethylene oxide and styrene, which damage DNA *in vitro* but do not seem to be carcinogenic in experimental animals, are carcinogenic in man.

A fourth reason for studying occupational cancer is that a carcinogen detected within the workplace may subsequently be found to have a different and possibly greater significance outside the workplace. In 1776, it was impossible for Pott to envisage that later a connection might be found between cancer of the scrotum in chimney sweeps and cancers of the respiratory tract associated with smoking tobacco.

The fifth and final reason which I give in support of studies to detect undisclosed carcinogens in the workplace may be the most important from the point of view of public health. As substances must be manufactured and formulated before they can be distributed, it is possible that a carcinogen—if sought for early enough—may be detected in the workplace in time to limit the exposure later on of a larger population of voluntary and involuntary consumers to a product or byproduct. Unfortunately, our current knowledge of the mechanisms of carcinogenesis is so deficient that we cannot depend solely upon *in vitro* and *in vivo* tests to pick out which chemicals are likely to be carcinogenic in man.

*How should we look for them?*

I propose to examine briefly four ways in which we might seek for undisclosed occupational carcinogens: by looking at patterns of cancer in time; by looking at geographical patterns; by studying the occupational histories of patients with cancer in retrospect; and by studying cancer risks of populations exposed to particular substances (such as formaldehyde, for example) compared with the risks of unexposed populations.

*Trends in time*

If we are to have the best chance of seeing temporal patterns of the incidence of cancer which point to factors in the environment, we need accurate measurements of the occurrence of new cases of cancer of each of the various organs and histological types of tumour, by age and for each sex, over a period of at least 3 decades. Furthermore, we need these to be undistorted by changes in classification and diagnosis. Such data are unfortunately not available.

Nevertheless, the data which are available in England and Wales are more comprehensive and cover more specific sites of cancer for a greater period of time than those of most other countries and are worthy of serious consideration. They are data about mortality, not incidence—with the disadvantages, and advantages, which mortality data have. We have reexamined the mortality in England and Wales in recent decades for 37 different sites of cancer. By applying a statistical model to mortality data collected in England over several decades we have attempted to separate the factors which influence the risk of death from cancer associated with year of birth (so-called generation effects) from factors associated with year of death (so-called period effects). There is truth, and also an element of over-simplification, in the statement that where there is a clear increase or decrease in the mortality of cancer in successive generations this is more likely to be due to a change in the environment to which successive generations have been exposed (changes for example in smoking or sexual

Fig. 1.—Generation and period effects based upon mortality from all neoplasms in persons under age 70 in England and Wales, 1950–78: (a) males, (b) females. These curves are produced by a statistical model which simultaneously calculates generation and period effects; where (i) a typical value of either is 1·0 and (ii) the values are proportional to the mortality attributed to the generation or period in question (Barrett, 1973).

habits) than to a change in diagnostic practice or treatment. The influence of changes in diagnostic practice or treatment is likely to affect different age-groups concurrently. On the other hand, increases or decreases in cancer mortality associated with year of death which affect most ages concurrently may be due to changes in diagnosis or treatment—*or* to environmental changes (like, for example, wartime rationing) which affect most of the population at the same time.

The analysis of mortality from all malignant tumours combined shows that both in men and in women in England and Wales the risk of death being certified as due to cancer has been declining in successive generations (Fig. 1). In men, the decline set in with the group born in the 5-year period centred around the year 1900, whereas for women the decline in mortality due to all cancers combined began with those born around 1925. If the principal tobacco-related cancers, namely cancers of the trachea, bronchus and lung, are excluded from the analysis the decline is observed to have begun much earlier. In so far as these patterns are attributable to environmental factors (in the widest sense of this term) and are not an artefact or due to improvements in treatment, this means that the resultant of the carcinogenic effect of all the agents (personal, social, chemical and physical) to which successive generations of men and women have been exposed in England has been diminishing. Similar patterns are obtained if persons dying over the age of 70 (where diagnosis is less exact than in younger people) are excluded.

Consideration of cancers of the various organs separately reveals that each has a different pattern. The analysis based on the model suggests, however, that a steadily increasing mortality in successive generations up to those born immediately before the Second World War has been comparatively rare, being present in only 5 of the 37 sites studied in each sex. The sites concerned are as follows: in both sexes there has been an increase, as defined, in the mortality from malignant tumours of brain and skin (including melanoma under this heading); the mortality of carcinoma of the breast and of the pancreas has increased in women; and the mortality from carcinoma of the kidney has increased in men. It should be pointed out that the analysis excluded deaths in children and in adults under 35 years of age, and a number of rare tumours.* Although the scale of the increases in successive generations detected in cancer of the organs mentioned above is slight (as compared for example with that seen in relation to lung cancer earlier in the century) it is worth considering the possibility that they represent the action of unfavourable environmental exposures or habits in successive generations, using the term "environmental" in its widest sense. For the remaining sites studied the generation effects are downwards or indeterminate.

In so far as they provide evidence that a carcinogenic agent (or agents) has not appeared in the environment on a scale sufficient to cause a sustained increase of the risk of cancer as a whole, or of most individual cancer sites, in successive generations of the general population during the period specified these curves are reassuring. There are, however, two reasons why they should not lead to complacency. The first is that it is possible for a carcinogen to appear and to increase the scale of its action during a period when there is a general decline in the mortality rate of cancer for the country as a whole. Asbestos, for example, which began to be imported into the United Kingdom on a substantial scale about the beginning of the century, must have been exerting its effect as a carcinogen on an increasing scale during the period before and after the Second World War (Doll, 1955; Acheson & Gardner, 1979). This was a period when the mortality from lung

* The absence of testis from this list is presumably due to limitation of the data to persons aged 35 and over (Davies, 1981).

cancer in men in England and Wales as a whole was already falling in successive generations.

The second proviso which must be made about the interpretation of time trends in cancer mortality is that, owing to the interval which elapses between exposure to carcinogens and the clinical appearance of related malignant tumours, these trends convey little about the carcinogenic impact of changes in the environment in the last 10–20 years. As far as the United States is concerned (and the trends in the United Kingdom are likely to be similar) it is since 1960 that the greatest increase in production of organic chemicals has taken place (Davies & Magee, 1979).

However, it does not necessarily follow that the number of people exposed to a chemical, let alone the number of people exposed to a biologically significant dose, increases in proportion to the amount produced. This depends, amongst other factors, on the degree to which controls on exposure levels have been put into effect during the corresponding period, and to the nature of the uses to which the substance is put.

The time trends for cancer mortality noted above are derived from the experience of the population of England and Wales as a whole. As people working in a particular occupation or industry represent only a small fraction of the whole, national mortality figures are unlikely to disclose the action of a carcinogen operating in the workplace—unless it has already escaped to the general environment. An analysis of time trends for different geographical areas would be more likely to be fruitful, and will be undertaken shortly, but even then dilution of the exposed population with what is generally a much larger population of unexposed persons and migration will limit sensitivity.

*Geographical patterns*

In the new MRC Unit of Environmental Epidemiology we have received on magnetic tape abstracts of all deaths which occurred in England and Wales during the period 1960–1979, a total of almost 12 million deaths. In so far as these data provide valid indicators of the actual diseases suffered by these men and women and their principal places of residence during life, it is possible that they will yield clues which will point to causes. First priority will be given to the publication of an atlas of mortality for England and Wales dealing with cancer along the lines of that recently produced in the United States. The scale of numbers at our disposal will enable us to include areas with smaller populations and rarer sites than was possible in Howe's classic atlas (Howe, 1970). For many sites of cancer we hope to map rates for each of more than 1,000 local authority districts.

In the preparatory work for the analysis of this material we have used the death rates for certain common cancers published by the Registrar General in his most recent area-mortality analysis for the period 1969–73 (OPCS, 1979). We have examined the geographical distribution of the standardized mortality ratios (SMRs) of these cancers in the 141 county boroughs and administrative counties in England and Wales. We have also studied the correlations between these SMRs and the distribution of the male workforce by industry according to place of work as recorded at the 1971 census (OPCS, 1975). As an example of this approach I will refer briefly to the pattern of mortality from bladder cancer in England and Wales.

During the period 1969–73, the mortality from bladder cancer in England and Wales was significantly raised ($P < 0{\cdot}01$) in either men or women or in both sexes in 10 of 83 county boroughs and 1 of 59 administrative counties. Using identical criteria it was found that the mortality from bladder cancer was significantly low in 7 administrative counties but in none of the county boroughs.

The general pattern therefore supports the relationship between mortality from bladder cancer and urban residence which has been demonstrated in the American geographical studies. This pattern is

FIG. 2.—Mortality from bladder cancer in England and Wales 1969–73, an analysis of 141 county boroughs and administrative counties. The map indicates 10 county boroughs and 1 administrative county with high mortality ($P < 0{\cdot}01$) in men, women or in both sexes.

partly due to occupational exposure, but may also be influenced by differences in smoking habits between town and country. The geographical pattern of the areas with a significantly high mortality from bladder cancer is striking, as all but 3 of the county boroughs (Gloucester, Brighton and Barrow) are in Yorkshire or Lancashire (Fig. 2). Huddersfield and Leeds have been centres of the dyestuffs industry for many years. Comparisons based on the distribution of industry in 1954 between the 10 county boroughs with significantly high SMRs and 10 county boroughs with the lowest SMRs suggested that, in addition to dyestuffs manufacture, more of the county boroughs with high rates had concentrations of rubber-, tyre- and paint-manufacturing industries but not of cable-making. We have at present no plausible explanation for the excess mortality from bladder cancer during 1969–73 in Brighton (in both sexes). The excess in men in Barrow has disappeared in more recent years. The excess in Brighton is being investigated further.

We have also studied the relationship of the SMRs from bladder cancer in the 141 areas to the distribution of the chemical industry, measured by the proportion of the total male workforce employed in 1971 (OPCS, 1975). A significant correlation was found between the mortality from bladder cancer in men and the proportion of employed men working in the chemical industry considered as a whole. When the different divisions of the chemical industry were considered separately there were correlations between the mortality from bladder cancer and employment in the dyestuffs and pigments division and with employment in the manufacture of soap and detergents (Table). Similar results were found in a study of bladder cancer in relation to the American chemical industry (Hoover & Fraumeni, 1975). Unlike the American study, we did not find correlations with the manufacture of pharmaceuticals or toilet preparations. There were no significant correlations in our material with the manufacture of general chemicals, paint, fertilizers or other chemicals.

TABLE.—*Relationship between SMRs (males, all ages) from bladder cancer* (1969–73) *and percentages of men in areas employed in chemical industry* (1971) *in* 141 *Couny Boroughs and Administrative Counties in England and Wales*

| Category within chemical industry | SMR from bladder cancer <80 | 80–99 | 100–119 | 120+ | Correlation coefficient |
|---|---|---|---|---|---|
| General chemicals | 0·7 | 0·9 | 1·2 | 0·5 | 0·4 |
| Pharmaceuticals | 0·2 | 0·3 | 0·3 | 0·3 | 0·9 |
| Toilet preparations | 0·05 | 0·07 | 0·04 | 0·1 | —0·03 |
| Paint | 0·2 | 0·1 | 0·09 | 0·2 | 0·04 |
| Soap and detergents | 0·01 | 0·1 | 0·07 | 0·2 | 0·19* |
| Synthetic resins | 0·2 | 0·4 | 0·4 | 0·1 | 0·03 |
| Dyestuffs and pigments | 0·03 | 0·05 | 0·2 | 0·4 | 0·30** |
| Fertilizers | 0·03 | 0·06 | 0·2 | 0·1 | 0·06 |
| Other chemicals | 0·2 | 0·3 | 0·1 | 0·2 | —0·09 |
| All categories | 1·7 | 2·3 | 2·7 | 2·0 | 0·16* |
| Number of areas | 21 | 59 | 44 | 17 | |

**$P<0{\cdot}01$ *$P<0{\cdot}05$.

It is too early to attempt any assessment of the usefulness or otherwise of a geographical approach as a means of providing clues for the identification of occupational carcinogens. However, it is reassuring that the method is at least sufficiently sensitive to demonstrate the effects of past exposure to the carcinogenic aromatic amines. The atlas of cancer mortality of the United States has already borne fruit (Mason & McKay, 1974). A concentration of lung cancer in coastal areas of the southeastern states has been found to be associated with asbestos used in shipyards in the Second World War (Blot *et al.*, 1978). The geographical clue derived from the atlas led to more rigorous field studies which demonstrated an association with an environmental factor. As is the case with time trends based on whole populations, sensitivity is reduced in geographical studies because the exposed populations represent a small proportion of the total populations contributing to the mortality rates.

*Case control studies*

The methods mentioned above have such major drawbacks in respect of dilution of the exposed populations, and because of uncontrolled confounding variables such as smoking, that the only justification for using them is that they can be used cheaply on a grand scale. At the other end of the scale of cost and practicability would be an attempt to accumulate detailed data about occupational exposure to chemicals, smoking and other relevant information in every person from the start of employment, and then to relate the accumulated information to the subsequent life risk of cancer. This is impracticable except in special populations such as professional chemists.

Two types of study which we are using fall between the two extremes mentioned above, and have in common the feature that they involve the collection of occupational data about individuals.

The "young cancer study", as it is called, sets out to collect data about occupation, industry and smoking for every male aged 18–54 in whom cancer was diagnosed during the years 1975–1980 inclusive. In the first instance, the survey is limited to those areas in England where there are concentrations of the chemical and steel industries. About 4,000 male patients or their relatives are being asked to complete a postal questionnaire. The distribution of occupations in men with cancer of a particular site will be compared with the distribution of occupations of men with all other types of cancer combined. In the second stage of the study an attempt will be made to list the substances to which the persons giving each job title are likely to have been exposed during the specified period.

Limiting the enquiry to men under the age of 55 will confer a number of advantages. Occupational histories will be shorter, better recollected and more likely to contain exposures to substances relevant to present-day and future experience. It is also likely that diagnosis will be more exact in this age group than in the elderly. Furthermore, for reasons about which there is a good deal of argument, the effects of carcinogens have often first been noted in relatively young subjects.

The advantages of this approach are that the occupational experience of a substantial sample of recently occurring cases of cancer can be used, and that results can be obtained quickly at low cost. In the light of experience we may subsequently include women, alter the range of age or site of tumour studied, or sample from other parts of the country. In any event, we hope the study will suggest hypotheses to be studied by more rigorous methods.

*Cohort studies*

Another approach is to start, not with the cancer patient as in the study just described and work backwards, but with a population of persons exposed to a particular substance and work forward in time to determine their cancer risk in relation to that of an unexposed population. If the records of men whose exposure to the substance in question began 20 or more years ago can be obtained, a realistic estimate of risk may be possible. This method is suitable in the study of substances for which, on the basis of *in vitro* tests or animal experiments or case reports, there is already a *prima facie* case for carcinogenic action in man.

Although more expensive than the studies already mentioned, the cost of such surveys is less than lifetime feeding experiments in animals. Furthermore, account should be taken of the fact that the result of a well conducted cohort study is directly relevant to man and does not require interspecies extrapolation. The limiting factor is not cost, but the practicability of identifying suitable industrial populations in which sufficient information is available, and gaining the necessary cooperation to follow them. The unit is currently involved in cohort studies of industrial populations exposed respectively to amosite asbestos, benzene, cadmium, epichlorhydrin, formaldehyde, glass fibre and styrene. To identify and study the other populations in which we are interested, for example, those exposed to ethylene oxide, lead, 1–4 dioxane, glycidaldehyde, amitrole, etc. we will need help from both sides of industry.

*Making old tools more serviceable*

Under this heading I wish to mention some ways in which, as an industrial society, we might move towards a strategy for the earlier discovery of carcinogens in the workplace although times are hard and likely to remain so. Our first priority should be to ensure that the unique facilities which are available in the United Kingdom, and which are the envy of other countries, should not be destroyed in the search for short-term economies. Prime among these is the facility for medical scientists to use the National Health Service Register at Southport (Adelstein, 1976. This register makes it possible to follow groups of persons exposed in industry, or defined in other ways, in respect of death and the occurence of cancer. A recent review has shown that at least 40 studies relevant to the causation of cancer are being carried out through this register (OPCS, 1981). Many of these relate to occupation, and the number is increasing. This work should be protected and extended. This register also contains detailed information about occupation and industry for those who were living in 1939. Information about mortality is now available for these men and women for more than 40 years. I believe that with ingenuity some of these data can be brought into service, and will be particularly useful in determining the late effects of carcinogenic substances in use before the War.

The National Cancer Register, which sets out to record all cases of cancer in England and Wales, has so far been of less value, because registration has been geographically uneven and data have been published after considerable delays. But it has become much more up-to-date recently and is potentially an important tool. Its organizers should be encouraged to initiate a drive to obtain data about main occupation and industry in more of its registered cases—starting with the regions with the highest proportion of primary manufacturing industry.

Two other relatively modest but important innovations can be recommended which will not be costly. At certification of death the registrar should be instructed to record, not only as at present the latest occupation of the deceased person, but the main lifetime occupation and the industry in which that occupation was held. The addition of industry would make it possible to distinguish, for example, between the various types of driver, fitter, machinist and process worker and would improve the sensitivity of the death certificate as an epidemiological tool. The scheme recently published by the Health and Safety Commission for the notification of new chemicals manufactured in quantities of 1 tonne or more should include provision that the records of persons exposed to them should be retained for a minimum of 30 years and made available for *bona fide* scientific study (HSE, 1981). It is also necessary to keep under review the question of whether the chemical industry, or even possibly primary manufacturing industry as a whole, should be required to retain a basic record identifying each member of their workforce for potential future linkage with records of cancer morbidity and mortality (Acheson, 1979). In the past it has been within primary manufacturing industry that most industrial carcinogens have been discovered (Cole & Goldman, 1975).

The suggestions that I have just mentioned are modest in cost, practicable, and seem an essential part of the housekeeping of an industrial society at the end of the 20th century. But there is one other issue which, unless it is settled satisfactorily, will serve to frustrate all innovations, and indeed will jeopardize future work in the identification of causes of human cancer in the workplace and elsewhere. This is the issue of the confidentiality of personal data, including medical records, and the rights of access of *bona fide* medical research workers to them. These issues are shortly to come before Parliament. It is essential that a solution should be found which will protect privacy without putting epidemiological research in chains, as has happened abroad. Until far more is known than at present about mechanisms of carcinogenesis, cases of cancer are unfortunately likely to occur due to exposure of men and women in the workplace. It is surely in the general interest that we should have the means to discover these at the earliest possible moment.

I am grateful to Mr Clive Osmond for the material shown in Fig. 1 and Mr Paul Winter for directing the computer work which formed the basis of Fig. 2 and the Table. Dr Martin Gardner made a number of helpful suggestions.

### REFERENCES

ACHESON, E. D. (1976) Nasal cancer in the furniture and boot and shoe manufacturing industries. *Prev. Med.*, **5**, 295.

ACHESON, E. D. (1979) Record linkage and the identification of long term environmental hazards. *Proc. R. Soc. Lond.* (*Biol.*), **205**, 165.

ACHESON, E. D., COWDELL, R. H. & HOLLES, B. (1970) Nasal cancer in the Northamptonshire boot and shoe industry. *Br. Med. J.*, i, 385.

ACHESON, E. D. & GARDNER, M. J. (1979) The ill effects of asbestos upon health. In *Asbestos, Vol. 2: Final Report of the Advistory Committee, Health and Safety Commission*. London: HMSO.

ADELSTEIN, A. M. (1976) Policies of the Office of Population Censuses and Surveys: Philosophy and constraints. *Br. J. Prev. Soc. Med.*, **30**, 1.

BARRETT, J. C. (1973) Age, time and cohort factors in mortality from cancer of the cervix. *J. Hyg.* (*Camb.*), **71**, 253.

BLOT, W. J., HARRINGTON, J. M., TOLEDO, A., HOOVER, R., HEATH, C. W. & FRAUMENI, J. F. (1978) Lung cancer after employment in shipyards during World War II. *N. Engl. J. Med.*, **299**, 620.

COLE, P. & GOLDMAN, M. B. (1975) *Persons at high risk of cancer: An approach to cancer aetiology and control.* Ed. Fraumeni. London: Academic Press. p. 167.

DAVIES, J. (1981) Testicular cancer in England and Wales: Some epidemiological aspects. *Lancet*, i, 928.

DAVIES, D. L. & MAGEE, B. H. (1979) Cancer and industrial chemical production. *Science*, **206**, 1356.

DOLL, R. (1955) Mortality from lung cancer in asbestos workers. *Br. J. Industr. Med.*, **12**, 81.

DOLL, R. (1979) Nutrition and cancer: A review. *Nutr. Cancer*, **1**, 35.

DOLL, R. (1975) Pott and the prospects for prevention. *Br. J. Cancer*, **32**, 263.

HEALTH & SAFETY EXECUTIVE (1981) *Consultative Document. Notification of New Substances.* 011 8834207.

HIGGINSON, J. & MUIR, C. S. (1979) Environmental carcinogenesis: Misconceptions and limitations to cancer control. *J. Natl Cancer Inst.*, **63**, 1291.

HOOVER, R. & FRAUMENI, J. F. (1975) Cancer mortality in counties with chemical industries. *Environ. Res.*, **9**, 196.

HOWE, G. M. (1970) *National Atlas of Disease Mortality in the United Kingdom.* London: Royal Geographical Society Medical Geography Committee.

MACBETH, R. G. (1965) Malignant disease of the paranasal sinuses. *J. Laryngol.*, **79**, 592.

MASON, T. J. & MCKAY, F. W. (1974) *Cancer Mortality by County, 1950–69.* Washington D.C.: Govt Printing Off.

OFFICE OF POPULATION CENSUSES AND SURVEYS (1979) *Area Mortality Tables. The Registrar General's decennial supplement for England and Wales, 1969–73.* Series DS No. 3.

OFFICE OF POPULATION CENSUSES & SURVEYS (1975) *Census of England and Wales, 1971.*

OFFICE OF POPULATION CENSUSES & SURVEYS (1975) *Census 1971. England and Wales. Economic Activity County Leaflets.* London: HMSO.

OFFICE OF POPULATION CENSUSES & SURVEYS (1981) *Report of the Advisory Committee on Cancer Registration, 1980* London: HMSO.


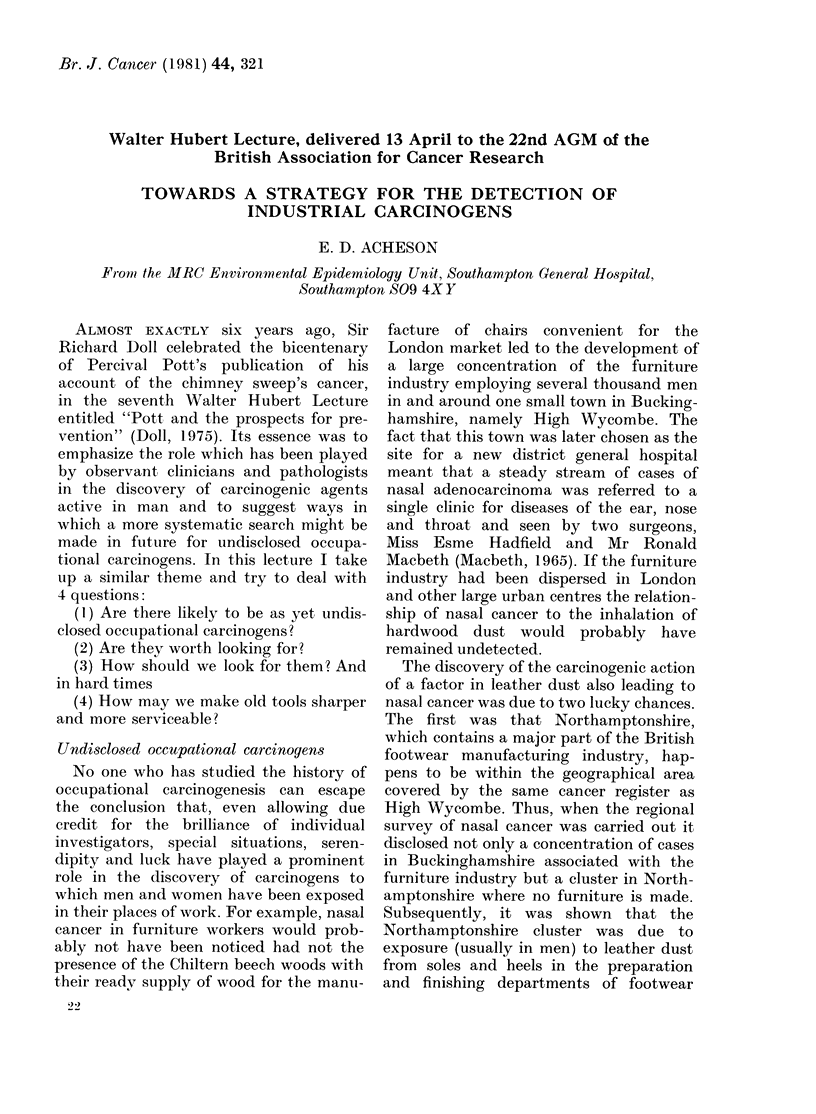

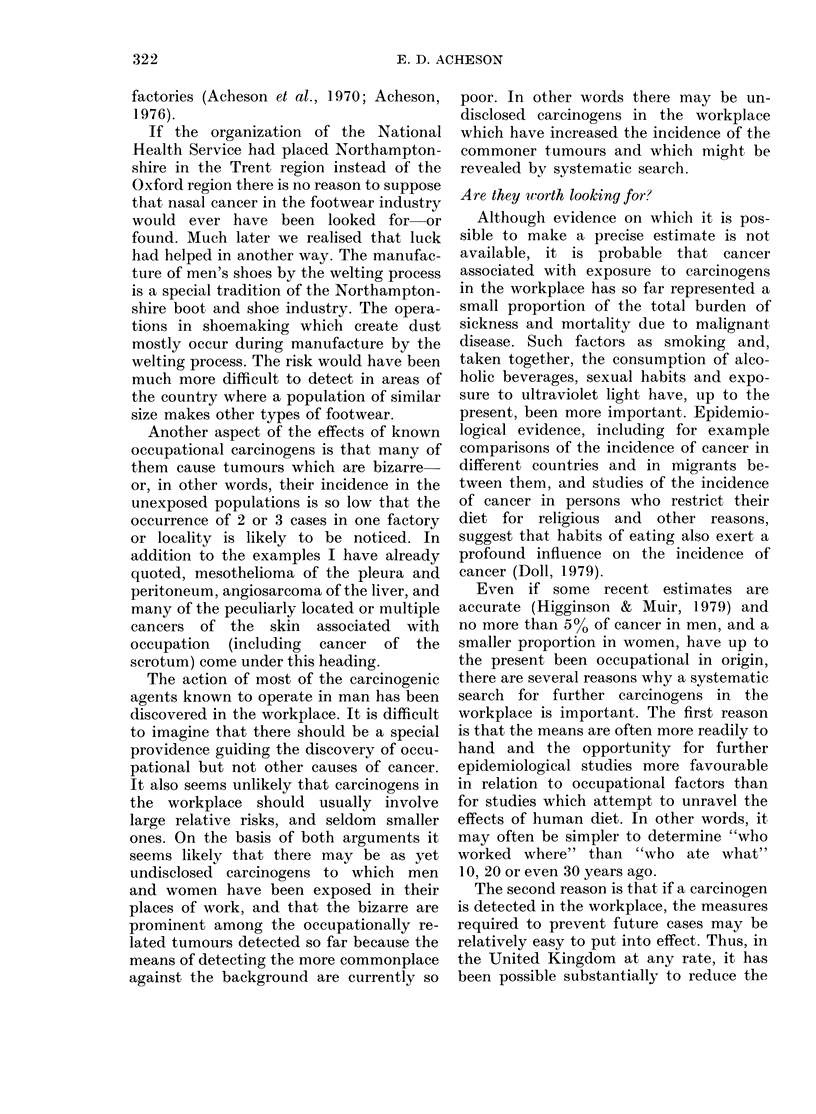

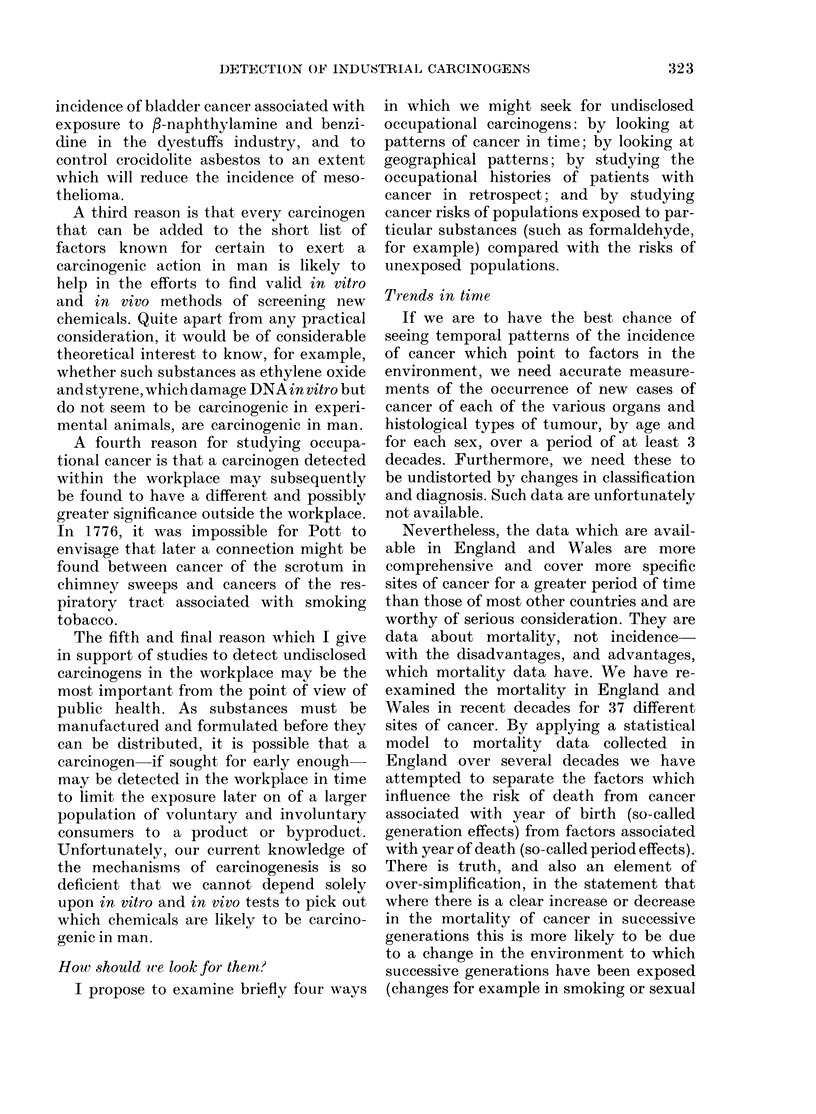

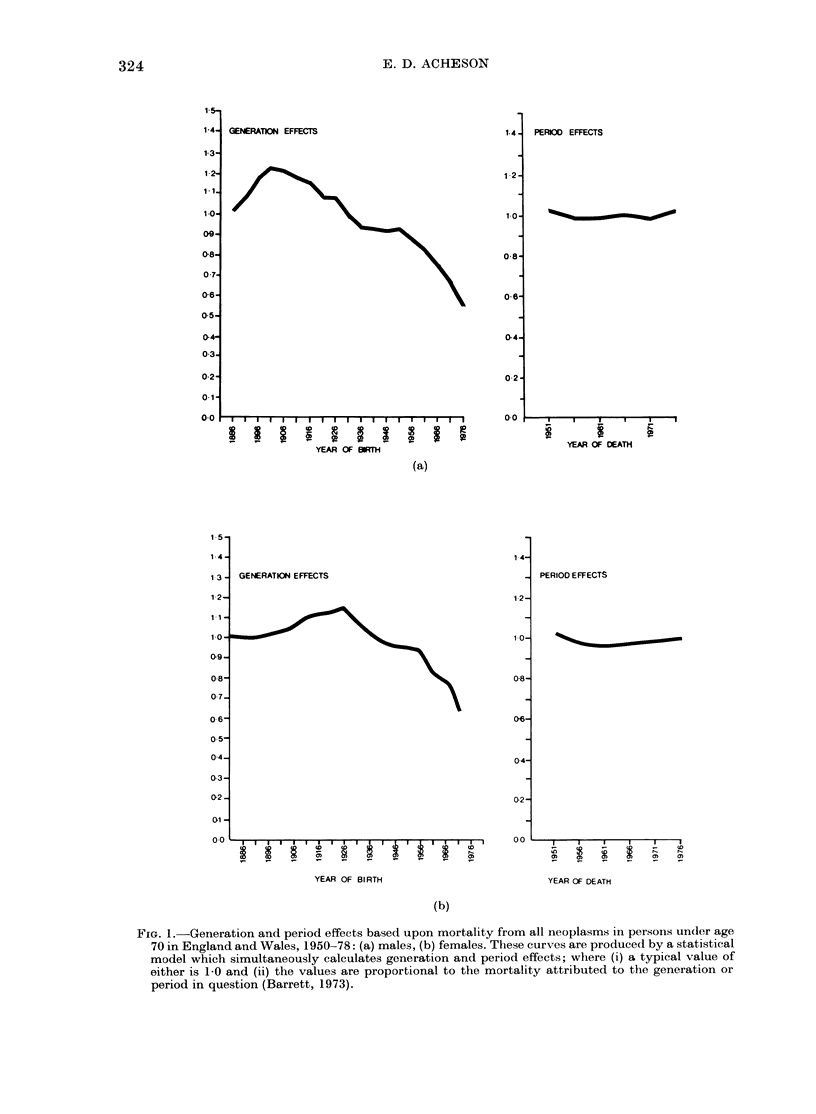

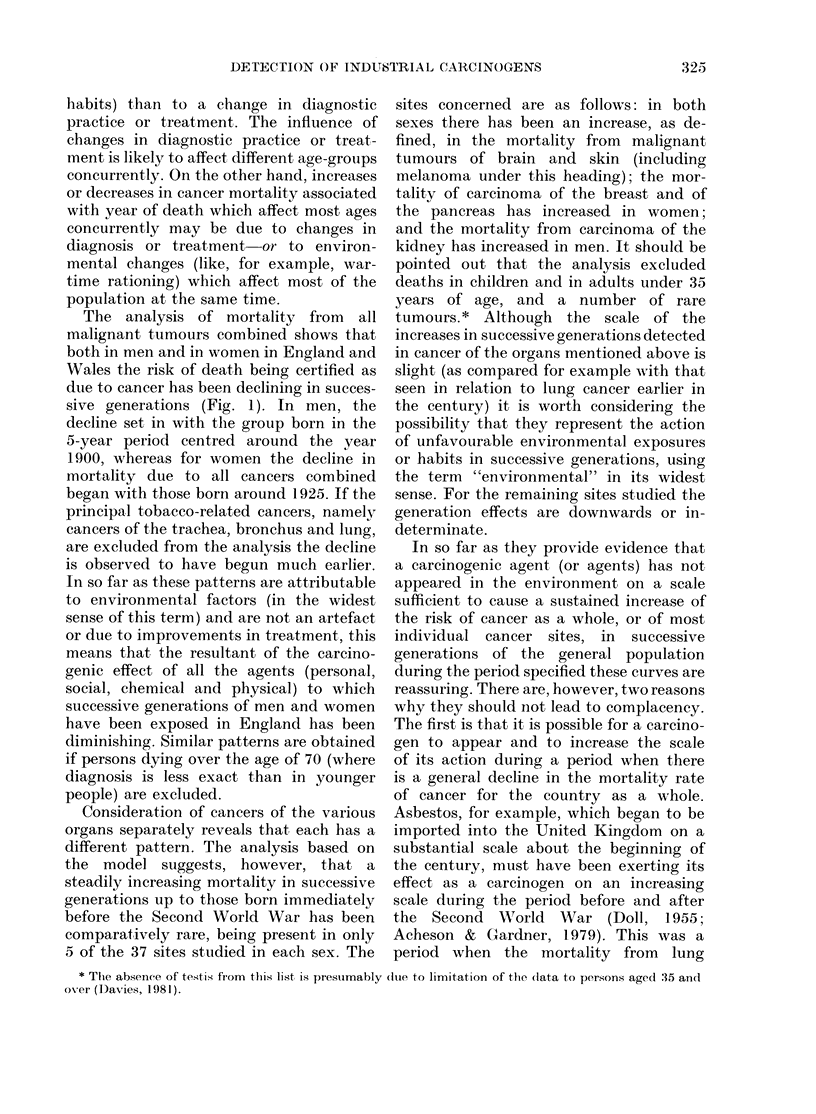

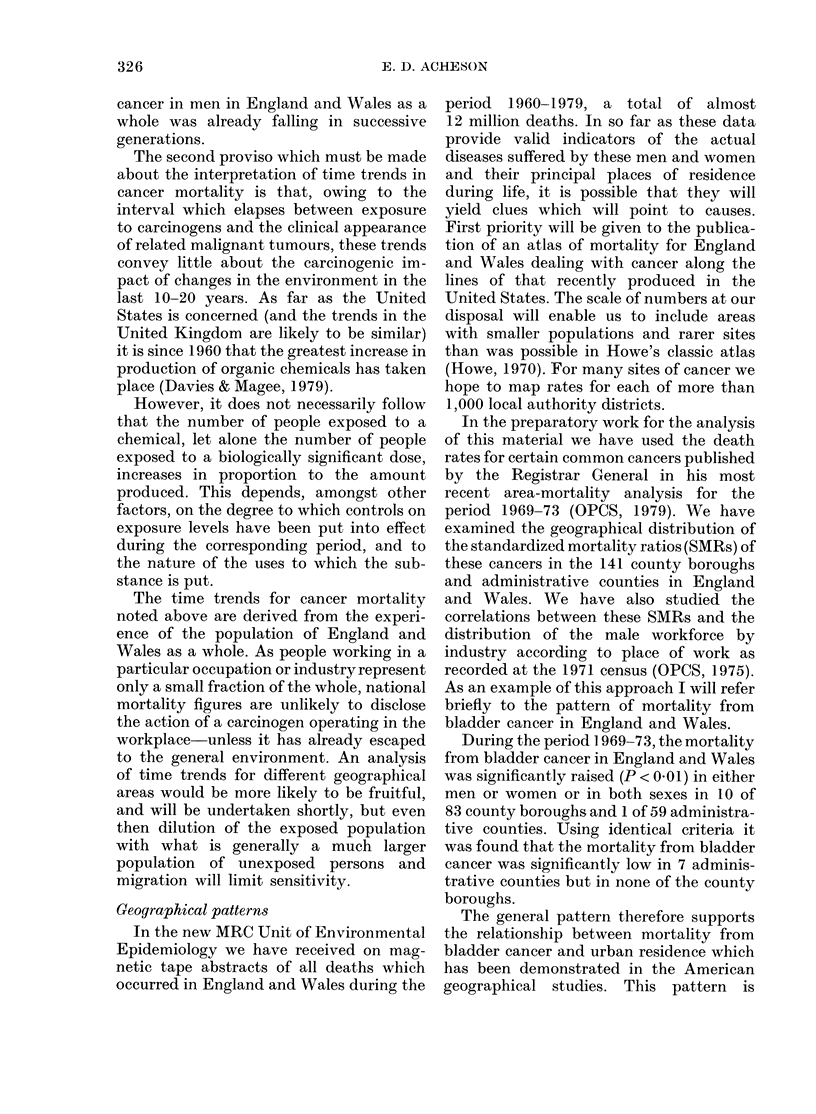

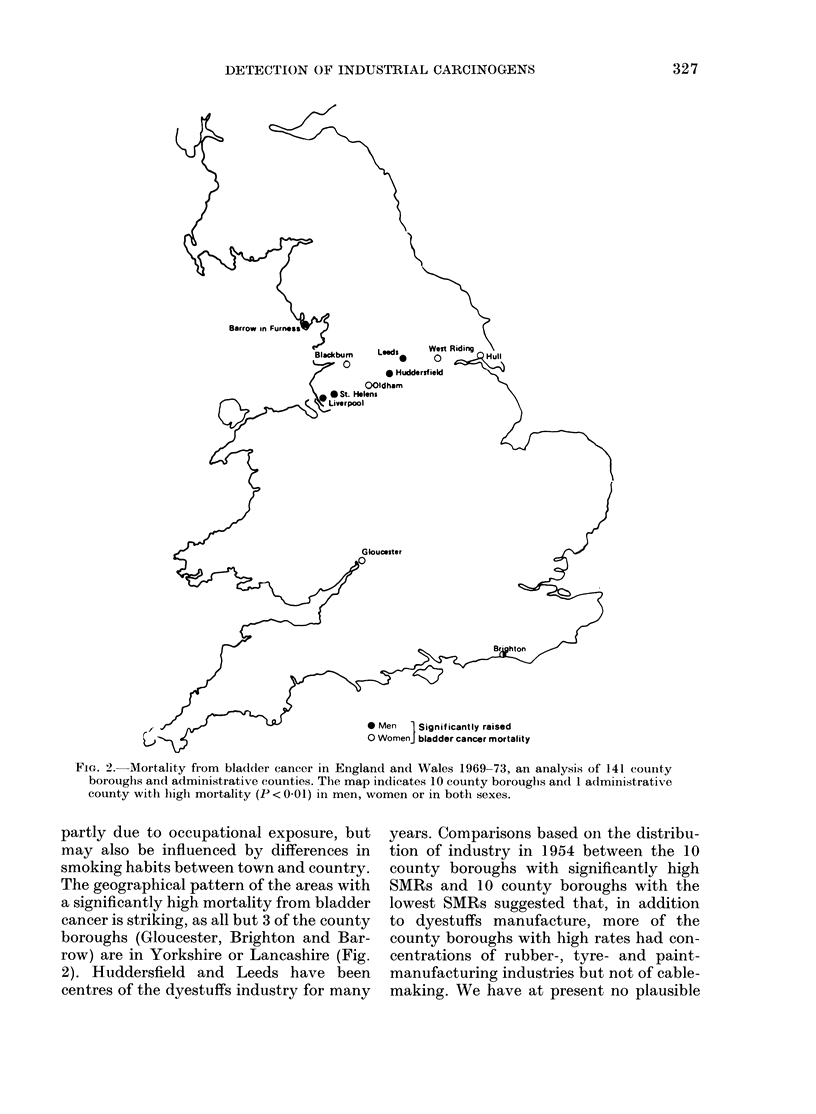

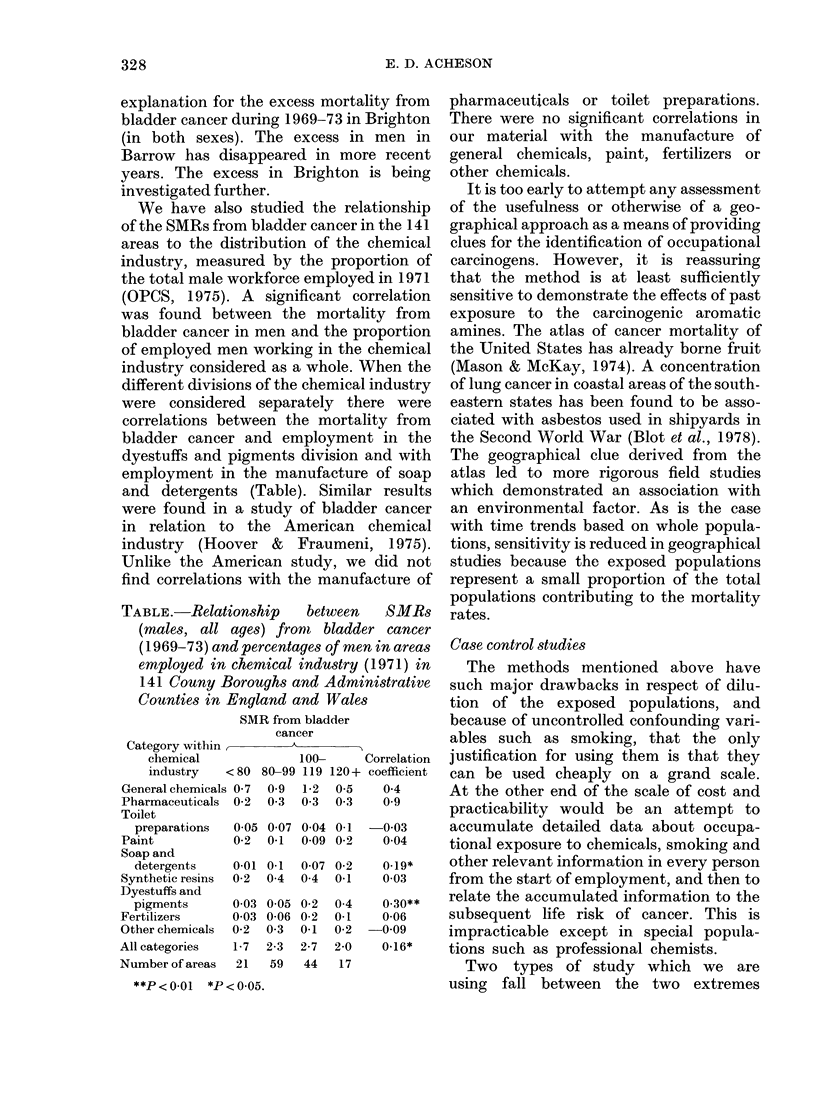

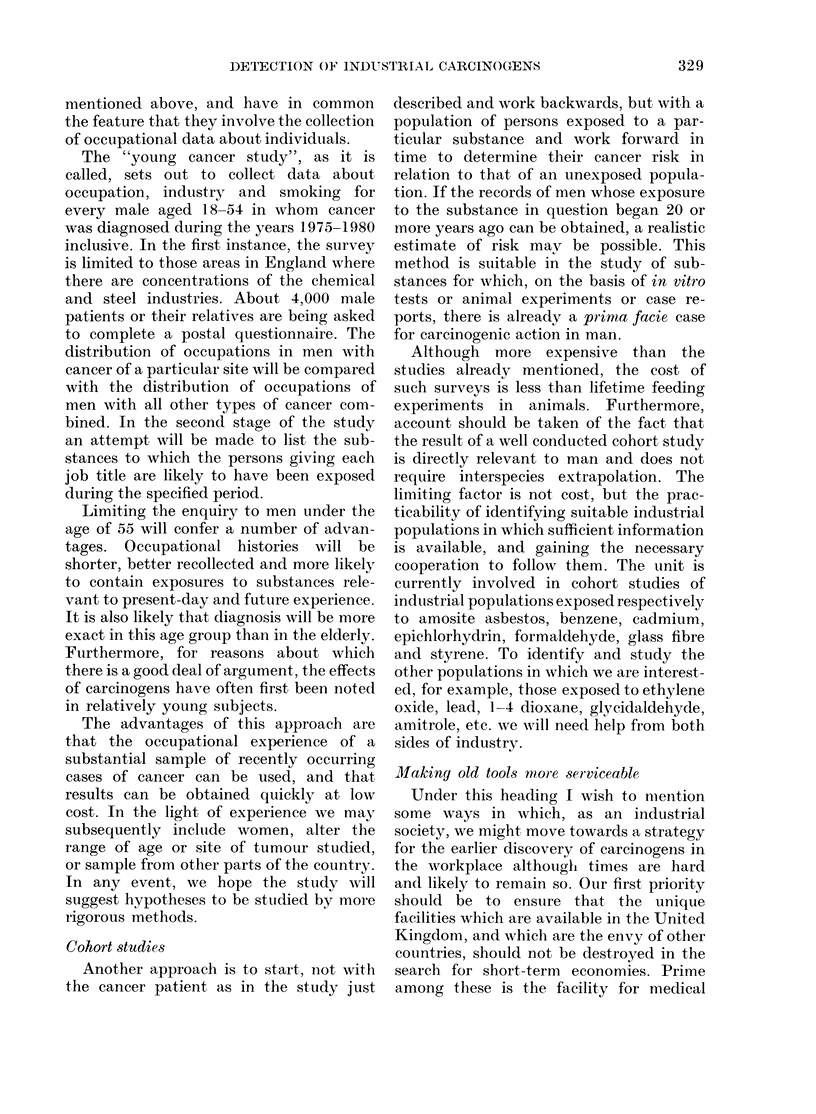

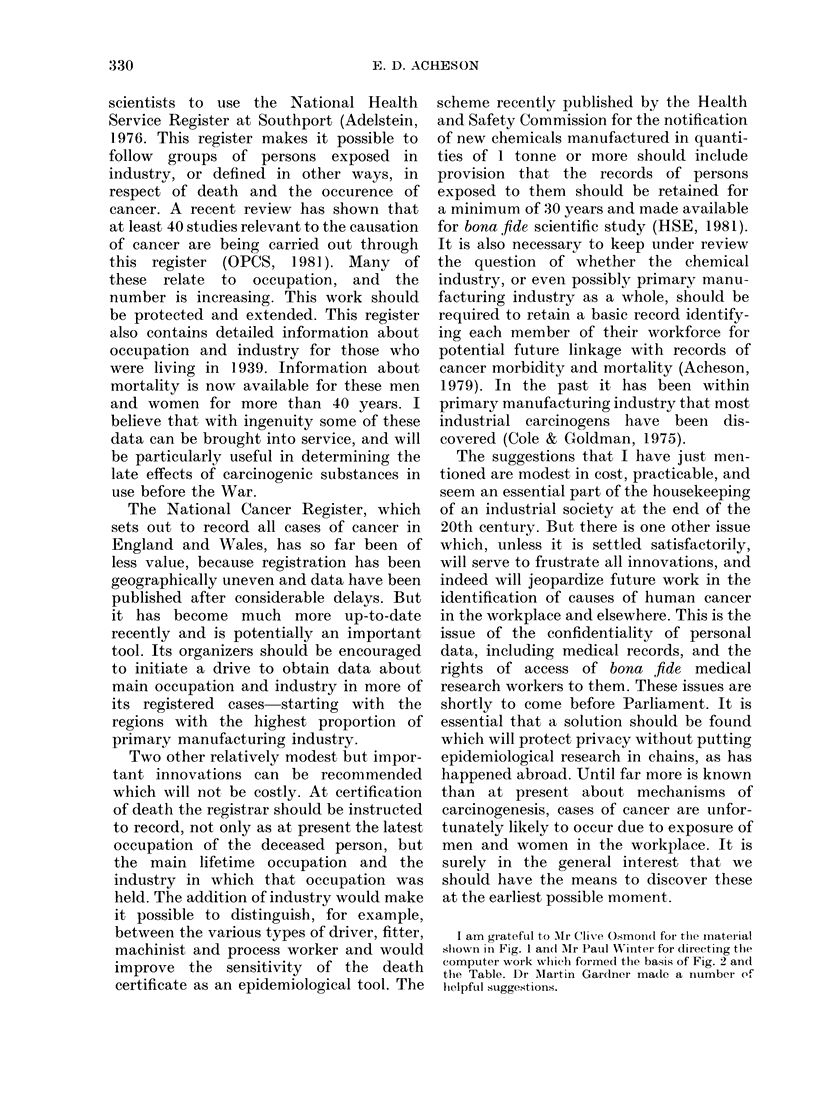

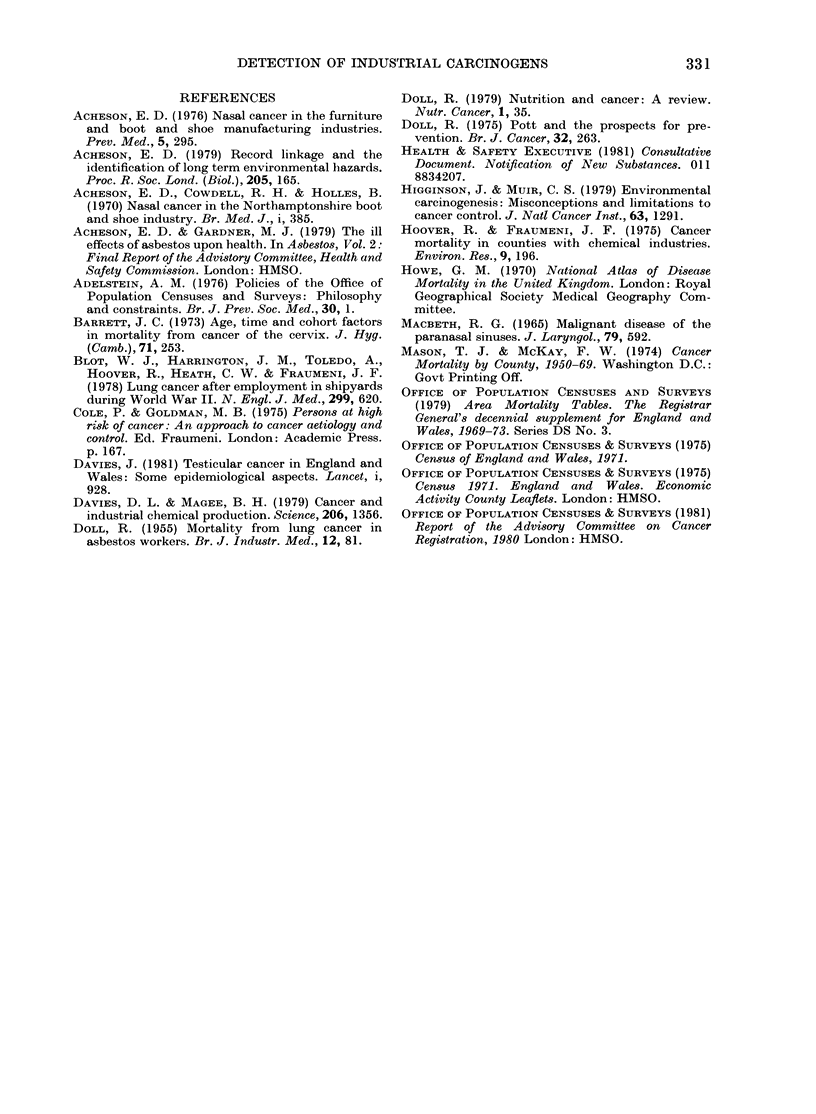


## References

[OCR_01085] Acheson E. D., Cowdell R. H., Jolles B. (1970). Nasal cancer in the Northamptonshire boot and shoe industry.. Br Med J.

[OCR_01075] Acheson E. D. (1976). Nasal cancer in the furniture and boot and shoe manufacturing industries.. Prev Med.

[OCR_01080] Acheson E. D. (1979). Record linkage and the identification of long-term environmental hazards.. Proc R Soc Lond B Biol Sci.

[OCR_01096] Adelstein A. M. (1976). Policies of the Office of Population Censuses and Surveys. Philosophy and constraints.. Br J Prev Soc Med.

[OCR_01101] Barrett J. C. (1973). Age, time and cohort factors in mortality from cancer of the cervix.. J Hyg (Lond).

[OCR_01106] Blot W. J., Harrington J. M., Toledo A., Hoover R., Heath C. W., Fraumeni J. F. (1978). Lung cancer after employment in shipyards during World War II.. N Engl J Med.

[OCR_01117] Davies J. M. (1981). Testicular cancer in England and Wales: some epidemiological aspects.. Lancet.

[OCR_01122] Davis D. L., Magee B. H. (1979). Cancer and industrial chemical production.. Science.

[OCR_01133] Doll R. (1975). Part III: 7th Walter Hubert lecture. Pott and the prospects for prevention.. Br J Cancer.

[OCR_01142] Higginson J., Muir C. S. (1979). Environmental carcinogenesis: misconceptions and limitations to cancer control.. J Natl Cancer Inst.

[OCR_01147] Hoover R., Fraumeni J. F. (1975). Cancer mortality in U.S. counties with chemical industries.. Environ Res.

[OCR_01158] MACBETH R. (1965). MALIGNANT DISEASE OF THE PARANASAL SINUSES.. J Laryngol Otol.

